# Design and synthesis of Fe_3_O_4_@SiO_2_@KIT-6@DTZ-Pd^0^ as a new and efficient mesoporous magnetic catalyst in carbon–carbon cross-coupling reactions

**DOI:** 10.1038/s41598-021-03485-4

**Published:** 2021-12-14

**Authors:** Zahra Moradi, Arash Ghorbani-Choghamarani

**Affiliations:** 1 Department of Chemistry, Faculty of Sciences, Ilam University, P.O. Box 69315516, Ilam, Iran; 2 Department of Organic Chemistry, Faculty of Chemistry, Bu-Ali Sina University, P.O. Box 6517838683, Hamedan, Iran

**Keywords:** Catalysis, Organic chemistry

## Abstract

In this paper, a new type of mesoporous material based on KIT-6 has been introduced. In this aim, magnetic Fe_3_O_4_ nanoparticles and mesoporous silica KIT-6 have been combined to obtain mesoporous MNPs. The prepared magnetic mesoporous catalyst has been applied in different carbon–carbon cross-coupling reactions including Mizoroki–Heck, Suzuki*–*Miyaura, and Stille reactions. This magnetic mesoporous compound is characterized by various techniques including FT-IR, BET, VSM, SEM, XRD, and TGA.

## Introduction

Mesoporous materials are solid and porous compounds with nanometer pore sizes and very high available surfaces area. These porous materials are made of two parts: cavities and walls. In the last few years, a different category of porous materials has been recognized and introduced to chemistry science, such as silica structures, metal oxides, silicon nanopores, carbon nanotubes, and porous carbon. The regular and porous silica structures were discovered in the 1990s^1–3^. High specific level, selectivity, shape, and size are the most significant characteristics of these materials, which convert them to critical materials with a wide range of applications such as catalysis, filtration and isolation etc.^4,5^. Major applications of nanopores in chemistry are their use in the manufacture of chemical sensors and application as a surface for the stabilization of chemical and biochemical catalysts. The development of these materials in the future depends on the manufacture of engineered and controlled porosity for the given applications^6–10^. Based on the size of the channel diameters, nanoporous materials are divided into three main categories: macroporous, mesoporous and microporous. The most important members of mesoporous, which are named as M41S family, include MCM-41, MCM-48, and MCM-50 mesopores^11,12^.

The KIT-6 structure is a relatively new structure of mesoporous silica, which has first been synthesized by Rio et al. in 2003 that has a cubic bilateral structure of Ia_3_d symmetry and cylindrical cavities and the advantage of this structure compared to other silica structures is the higher cavities volumes^13,14^. This three-dimensional silica grid can be a suitable host for various components, which can easily enter the cavity and spread out. KIT-6 has been recognized with wide applications including electrical, catalytic, and isolation applications^15–18^.

One of the most important chemists’ achievements is the discovery of the carbon–carbon coupling reactions in the presence of a complex containing transition metal such as copper, palladium, nickel, and iron. Among transition metals complexes, palladium is one of the best choices, due to its simple complex preparation, high activity, and selectivity in coupling reactions^19–24^. In organic chemistry science, a variety of coupling reaction systems has been discovered, designed, and introduced: Heck reaction^25^, Suzuki reaction^26^, Negishi reaction^27^, Stille reaction^28^, Sonogashira reaction^25^, Kumada coupling^26^, Hiyama coupling reaction^27^, and Buchwald–Hartwig reaction^29^. Among them, Stille (coupling reaction of a tin organic compound with an electro-friendly organic compound)^30^, Suzuki (coupling reaction of a phenylboronic acid derivative with various aryl halides)^31^, and Heck reaction (coupling reaction of aryl of various halides with alkenes)^32^ are main and generally applicable methods for the construction of carbon–carbon bonds^33,34^. Herein we have synthesized a new immobilized palladium complex on the modified magnetic mesoporous material (Fe_3_O_4_@SiO_2_@KIT-6@DTZ-Pd^0^) that acts as a versatile catalyst in cross-coupling reactions.

## Result and discussion

### Preparation and characterization of Fe_3_O_4_@SiO_2_@KIT-6@DTZ-Pd^0^

**Figure 1:**
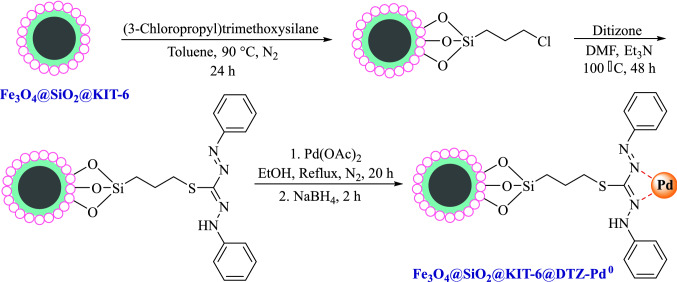
Stepwise preparation of Fe_3_O_4_@SiO_2_@KIT-6@DTZ-Pd^0^.

For the synthesis of Fe_3_O_4_@SiO_2_@KIT-6@DTZ-Pd^0^ catalyst, first Fe_3_O_4_ magnetic nanoparticles were synthesized by co-precipitation method^35^. Subsequently, it was coated by KIT-6 to synthesize Fe_3_O_4_@SiO_2_@KIT-6 nanoparticles. In the next step, obtained Fe_3_O_4_@SiO_2_@KIT-6 modified by (3-chloropropyl) triethoxysilane. Finally, to obtain the final catalyst Fe_3_O_4_@SiO_2_@KIT-6-nPrNH_2_ condensed with dithizone followed by coordination with palladium as outlined in Fig. 1.

After designing and fabricating Fe_3_O_4_@SiO_2_@KIT-6@DTZ-Pd^0^, the structure of this magnetic mesoporous material is characterized by various techniques.

**Figure 2:**
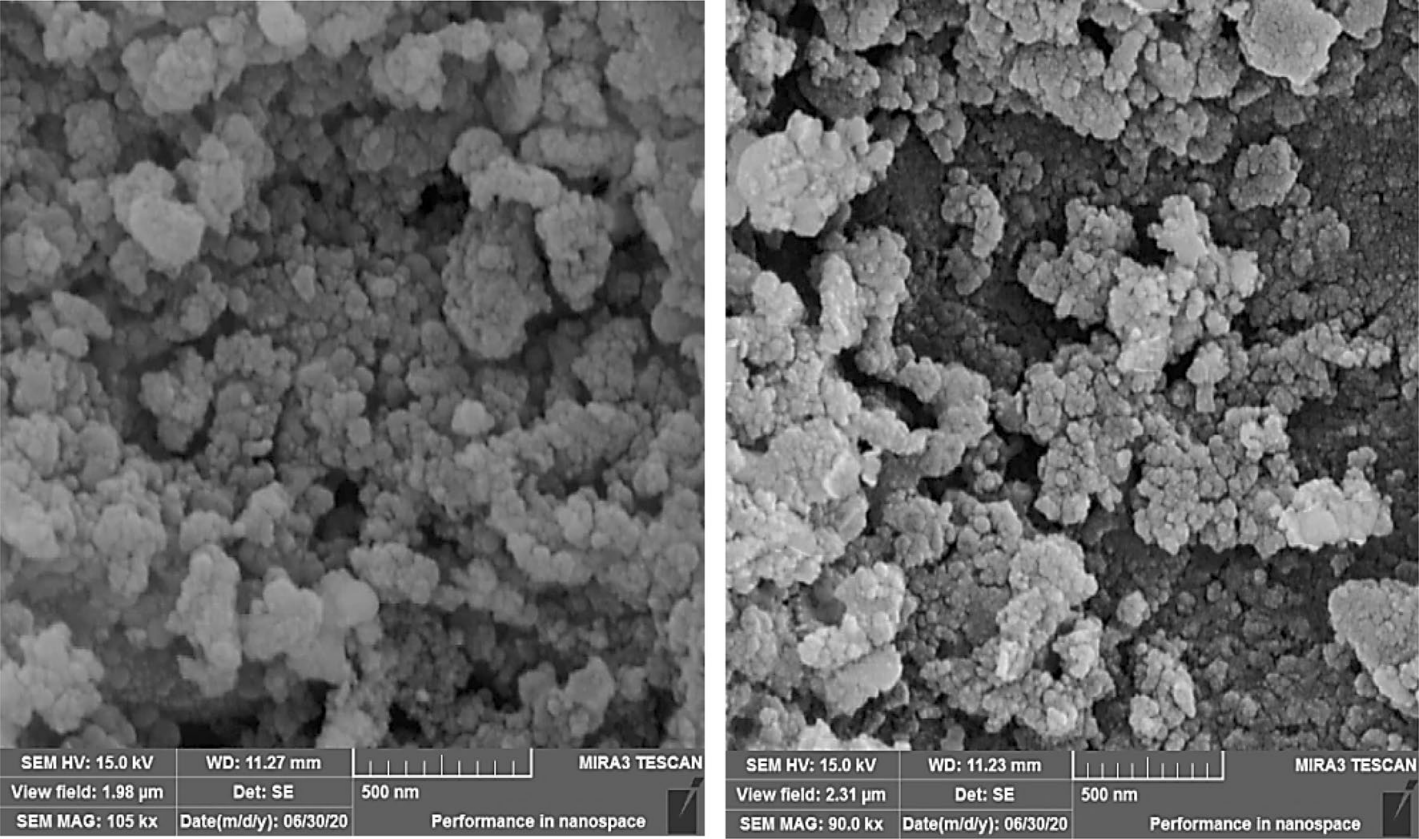
SEM images of the Fe_3_O_4_@SiO_2_@KIT-6@DTZ-Pd^0^ at different magnification.

To consider the morphology of prepared magnetic mesoporous material, SEM (scanning electron microscopy) was applied. As it can be seen in Fig. 2, Fe_3_O_4_@SiO_2_@KIT-6@DTZ-Pd^0^ particles are spherical with nano-sized particles.

**Figure 3:**
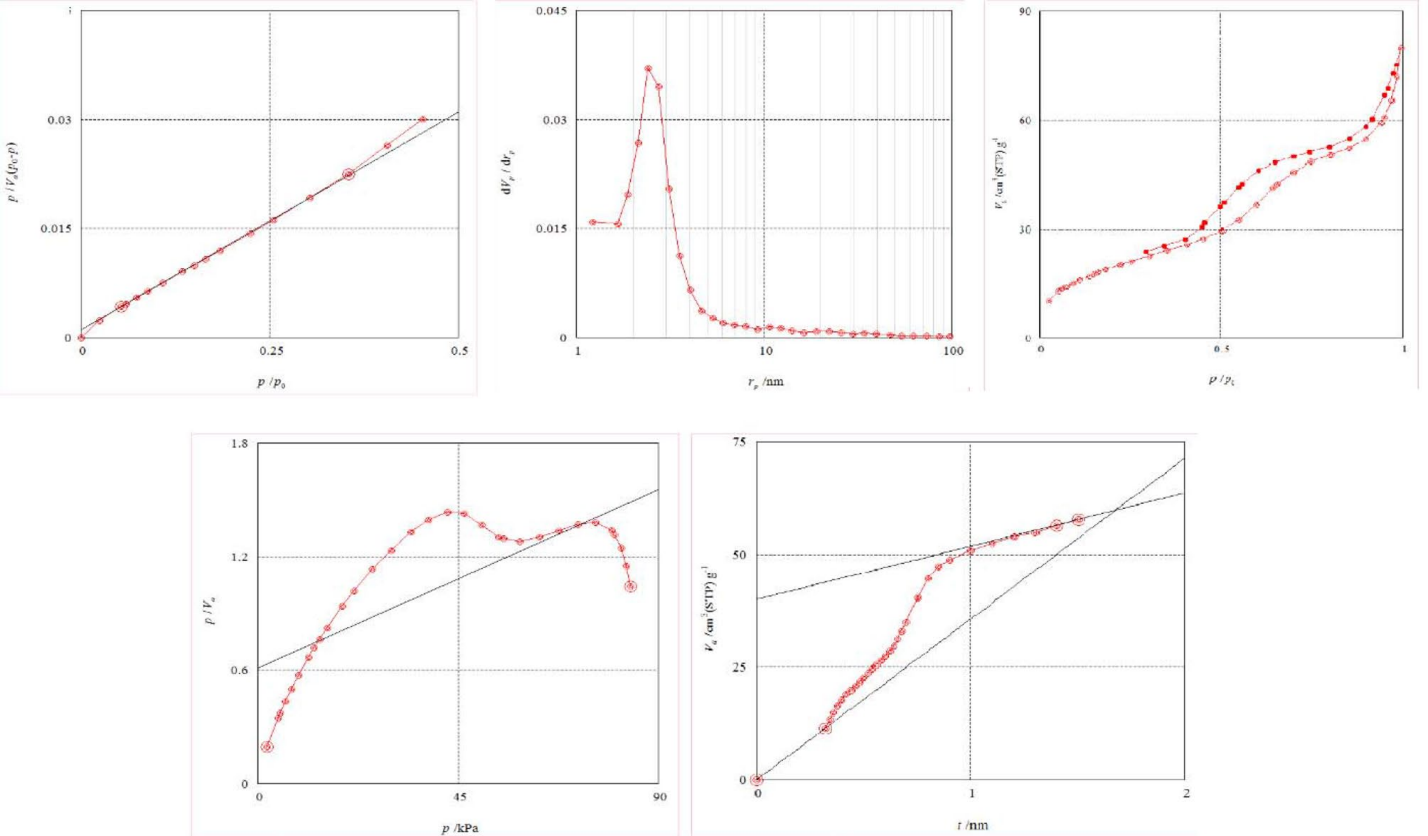
N_2_ adsorption–desorption isotherms of Fe_3_O_4_@SiO_2_@KIT-6@DTZ-Pd^0^.

Porosity analysis of Fe_3_O_4_@SiO_2_@KIT-6@DTZ-Pd^0^ studied by nitrogen absorption–desorption technique. Based on this analysis, the specific surface area of Fe_3_O_4_@SiO_2_@KIT-6@DTZ-Pd^0^ (a_SBET_) was 71.477 m^2^/g, its monolayer capacity (V_m_) was 16.422 cm^3^ (STP) g^−1^ and the total pore volume of the prepared magnetic mesoporous compound was 0.1188 cm^3^/g. The specific surface area was calculated by the Langmuir isotherm. Langmuir isotherm (Fig. 3) illustrated a_s,lang_ = 415.1 m^2^/g and V_m_ = 95.372 cm^3^ (stp) g^−1^, respectively. Furthermore, the calculations related to BJH diagram from the adsorption and desorption branch of the nitrogen adsorption curve indicated that the pore sizes in these compounds were the same and the average pore diameter of Fe_3_O_4_@SiO_2_@KIT-6@DTZ-Pd^0^ is 2.41 nm.

To measure the exact amount of loaded Pd on the surface of Fe_3_O_4_@SiO_2_@KIT-6@DTZ nanoparticles, induced coupled plasma (ICP) spectroscopy technique was used, which based on this technique, the exact amount of Pd is 1.069 × 10^–3^ mol/g.

**Figure 4:**
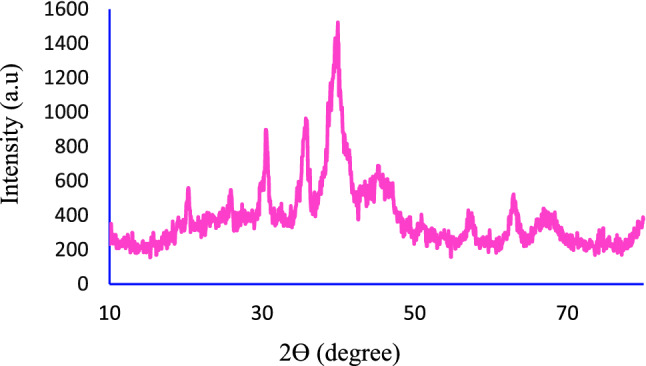
The XRD pattern of the Fe_3_O_4_@SiO_2_@KIT-6@DTZ-Pd^0^.

The XRD pattern of Fe_3_O_4_@SiO_2_@KIT-6@DTZ-Pd^0^ nanoparticles is displayed in Fig. 4. As it can be seen, the XRD pattern of synthesized nanoparticle shows characteristic peaks at 2θ = 25.86°, 30.46°, 35.71°, 43.46°, 53.86°, 57.01°, 62.86, and 74.16° that are related to Fe_3_O_4_ and the extent peak appearing in 2θ of 20.26° are related to the presence of a silica layer around the nanoparticles. The corresponding peaks at 2θ = 39.91°, 45.16° and 66.86° 2θ is correspondence to Pd particles. These pieces of evidence show that Pd is well stabilized on the nanocatalyst^36,37^.

**Figure 5:**
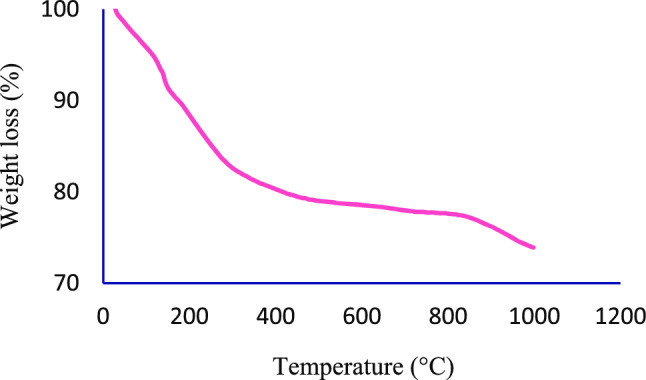
TGA thermogram of the Fe_3_O_4_@SiO_2_@KIT-6@DTZ-Pd^0^.

The TGA diagram for magnetic mesoporous material (Fe_3_O_4_@SiO_2_@KIT-6@DTZ-Pd^0^) is shown in Fig. 5. Based on this analysis, the first weight loss under 200 °C (about 10%) is related to the evaporation of physically adsorbed solvents and water. The second weight loss, which is about 9.5%, is between 200 to 800 °C related to the removal of organic moieties on the surface of magnetic mesoporous support. The final weight loss is related to the phase change of Fe_3_O_4_@SiO_2_@KIT-6@DTZ-Pd^0^ nanoparticles.

**Figure 6:**
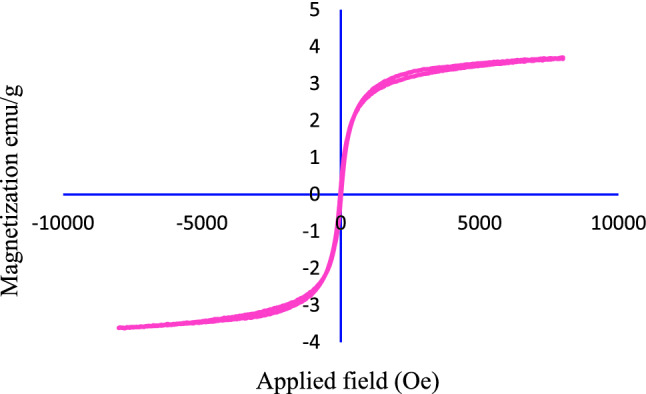
Magnetization curves for Fe_3_O_4_@SiO_2_@KIT-6@DTZ-Pd^0^ at room temperature.

The magnetic properties of the synthesized Fe_3_O_4_@SiO_2_@KIT-6@DTZ-Pd^0^ were investigated by VSM analysis. As displayed in Fig. 6, the magnetic property of nanocatalyst is 3.67 emu/g, which reflects this fact, the nanoparticle surface is coated with SiO_2_ and organic groups. Nevertheless, by applying an external magnetic field, the catalyst can be easily separated from the reaction mixture.

**Figure 7:**
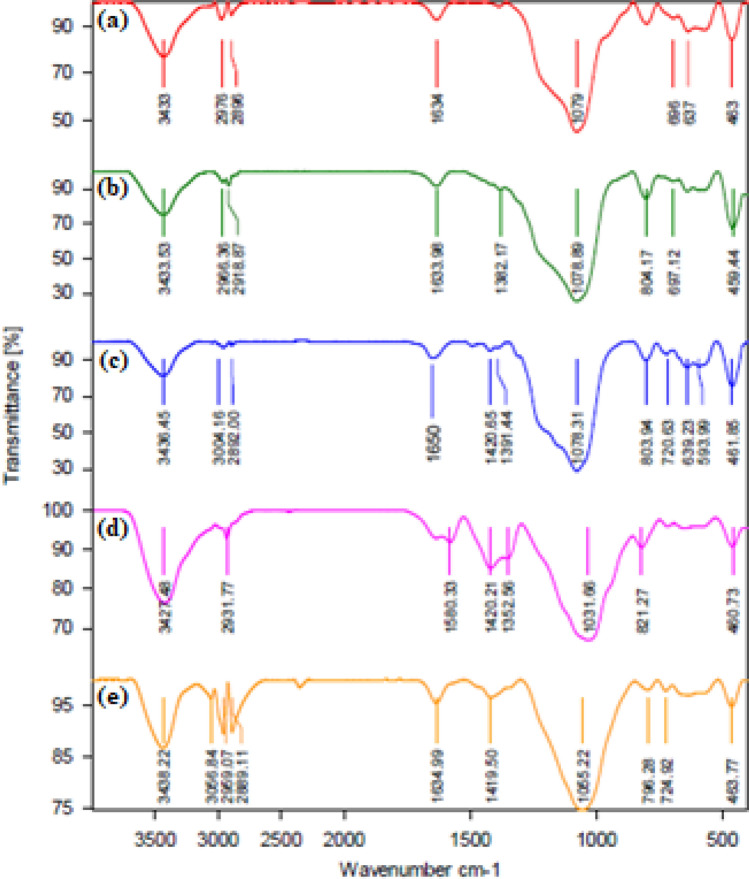
The FT-IR spectra of Fe_3_O_4_@SiO_2_@KIT-6 (**a**), Fe_3_O_4_@SiO_2_@KIT-6@CPTES (**b**), Fe_3_O_4_@SiO_2_@KIT-6@DTZ (**c**), Fe_3_O_4_@SiO_2_@KIT-6@DTZ-Pd^0^ (**d**) and recovered Fe_3_O_4_@SiO_2_@KIT-6@DTZ-Pd^0^ (**e**).

The FT-IR spectra of Fe_3_O_4_@SiO_2_@KIT-6 (**a**), Fe_3_O_4_@SiO_2_@KIT-6@CPTES (**b**), Fe_3_O_4_@SiO_2_@KIT-6@DTZ (**c**), Fe_3_O_4_@SiO_2_@KIT-6@DTZ-Pd^0^ (**d**) and recovered Fe_3_O_4_@SiO_2_@KIT-6@DTZ-Pd^0^ (**e**) are shown in Fig. 7. The FT-IR spectra of Fe_3_O_4_@SiO_2_@KIT-6 (Fig. 7a) indicated several bands at 463 cm^−1^, 637 cm^−1^, which is related to stretching vibration of Fe–O bond, and a sharp peak at 1079 cm^−1^ belong to stretching vibration of Si–O–Si bond, two peaks at 1634 cm^−1^ and 3433 cm^−1^ related to bending vibration and stretching vibration OH groups, respectively. Several peaks about 2900 cm^−1^ are related to C–H stretching vibration and the bending vibration for Si–O–Si bond appears at 1078 cm^−1^ in the IR spectra of Fe_3_O_4_@SiO_2_@KIT-6@CPTES (Fig. 7b). In the next spectra (Fig. 7c) the observed peaks at 3436 cm^−1^ and 1650 cm^−1^ are related to N–H and C=N bonds, also the observed peak at 1391 cm^−1^ is belongs to the stretching vibration of C=S bond. The shifting of C=N peak from 1650 to 1634 cm^−1^ in IR spectra of Fe_3_O_4_@SiO_2_@KIT-6@DTZ-Pd (Fig. 7d) indicating that palladium was successfully coordinated to dithizone. The FT-IR spectra of recovered Fe_3_O_4_@SiO_2_@KIT-6@DTZ-Pd^0^ (Fig. 7e), after the four times recovery, do not show any significant change with the fresh catalyst.

### Catalytic studies

#### Mizoroki–Heck cross-coupling reaction

After the characterization of the prepared magnetic mesoporous material, the catalytic activity of this compound was studied in carbon–carbon bond formation reactions.

Initially, the catalytic activity of Fe_3_O_4_@SiO_2_@KIT-6@DTZ-Pd^0^ was examined in Heck reaction. To obtain optimal conditions for this C–C cross-coupling reaction, the reaction of iodobenzene and butyl acrylate was investigated as a model reaction. The reaction was examined in the presence of various solvents such as PEG, DMF, DMSO, CH_3_CN, different bases (KOH, NaOH, Na_2_CO_3_, Li_2_CO_3_), and different values of catalysts (4, 5, and 6 mg) at various temperature conditions.

The highest yield of product (95%) was obtained in PEG as the solvent, 3 mmol of K_2_CO_3_ as the base and 5 mg of catalyst at 100 °C (Table 1).

**Table 1:** Optimization conditions of Mizoroki–Heck cross-coupling reaction of iodobenzene and butyl acrylate. Significant values are in bold.

Entry	Solvent	Base	Base (mmol)	Catalyst (mg)	Temperature (°C)	Time (min)	Yield (%)^a^
**1**	**PEG**	**K**_**2**_**CO**_**3**_	**3**	**5**	**100**	**35**	**95**
2	DMSO	K_2_CO_3_	3	5	100	35	67
3	DMF	K_2_CO_3_	3	5	100	35	65
4	CH_3_CN	K_2_CO_3_	3	5	Reflux	35	59
5	PEG	KOH	3	5	100	35	53
6	PEG	Na_2_CO_3_	3	5	100	35	76
7	PEG	Li_2_CO_3_	3	5	100	35	61
8	PEG	NaOH	3	5	100	35	45
9	PEG	K_2_CO_3_	3	6	100	35	96
10	PEG	K_2_CO_3_	3	4	100	35	54
11	PEG	K_2_CO_3_	1.5	5	100	35	63
12	PEG	K_2_CO_3_	3	5	120	35	96
13	PEG	K_2_CO_3_	3	5	80	35	65
14	PEG	K_2_CO_3_	3	5	60	35	33

^a^Isolated yield.

**Scheme 1:**
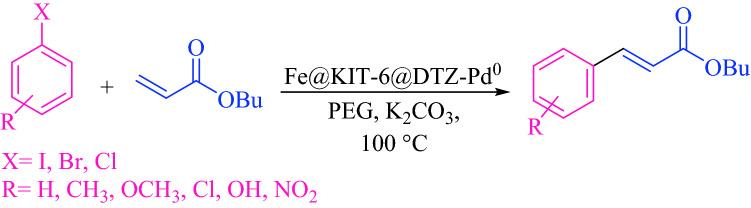
Mizoroki–Heck cross-coupling reaction.

To develop the efficiency of described catalyst a variety of aryl halides reacted with butyl acrylate under obtained optimal reaction conditions (Scheme 1).

The results are summarized in Table 2. As it can be seen, aryl iodides are not significantly different from aryl bromides and chlorides in terms of the reaction yields, and only the reaction time with aryl bromides and aryl chlorides is slightly longer.

**Table 2:** Mizoroki–Heck cross-coupling reaction in the presence of a catalytic amount of Fe_3_O_4_@SiO_2_@KIT-6@DTZ-Pd^0^.

Entry	X	R	Time (min)	Yield (%)^a^	M.P (°C)	TON	TOF (h^−1^)
1	I	H	35	95	Oil^38^	177.73	304.85
2	I	4-CH_3_	20	92	Oil^38^	172.123	516.88
3	I	4-OCH_3_	15	90	Oil^30^	168.381	673.52
4	Br	4-OCH_3_	40	90	Oil^38^	168.381	252.825
5	Br	4-CH_3_	75	87	Oil^38^	162.768	130.215
6	Br	H	130	85	Oil^38^	159.027	61.164
7	Br	4-Cl	145	89	Oil^39^	166.510	68.92
8	Br	4-NO_2_	180	85	60–61^38^	159.027	53.009
9	Cl	H	270	79	Oil^31^	147.801	32.844

^a^Isolated yield.

### Suzuki*–*Miyaura cross-coupling reaction

To consider more activity of Fe_3_O_4_@SiO_2_@KIT-6@DTZ-Pd^0^ as the catalyst, the activity of the described catalyst was studied in the Suzuki cross-coupling reaction. The optimization experiment initiated the reaction of iodobenzene with phenylboronic acid in different conditions as a model substrate. Reaction conditions including base, solvent, temperature, the amount of catalyst were screened and the results are summarized in Table 3. The results demonstrated that the highest efficiency was achieved in Ethanol as the solvent, K_2_CO_3_ (2.5 mmol), and was 5 mg of catalyst at 75 °C.

**Table 3:** Optimization of Suzuki–Miyaura cross-coupling reaction of iodobenzene and phenylboronic acid. Significant values are in bold.

Entry	Solvent	Base	Base (mmol)	Catalyst (mg)	Temperature (°C)	Time (min)	Yield (%)^a^
1	PEG	K_2_CO_3_	2.5	5	75	12	90
**2**	**EtOH**	**K**_**2**_**CO**_**3**_	**2.5**	**5**	**75**	**12**	**95**
3	DMF	K_2_CO_3_	2.5	5	75	12	48
4	CH_3_CN	K_2_CO_3_	2.5	5	75	12	59
5	EtOH	KOH	2.5	5	75	12	89
6	EtOH	Na_2_CO_3_	2.5	5	75	12	71
7	EtOH	NaOH	2.5	5	75	12	78
8	EtOH	K_2_CO_3_	3	5	75	10	95
9	EtOH	K_2_CO_3_	2	5	75	12	81
10	EtOH	K_2_CO_3_	2.5	7	75	10	93
11	EtOH	K_2_CO_3_	2.5	6	75	12	92
12	EtOH	K_2_CO_3_	2.5	4	75	12	69
13	EtOH	K_2_CO_3_	2.5	5	60	12	76
14	EtOH	K_2_CO_3_	2.5	5	45	12	51

^a^Isolated yield.

**Scheme 2:**
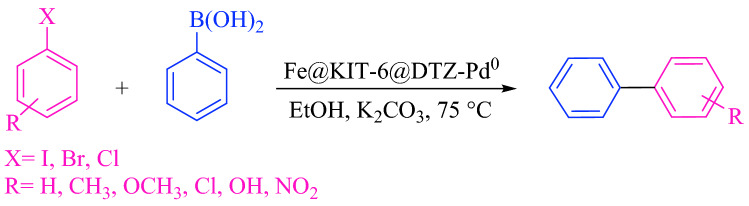
Suzuki–Miyaura cross-coupling reaction.

The optimal conditions were applied for the reaction of a wide variety of aryl halides with phenylboronic acid (Scheme 2); the results are presented in Table 4.

**Table 4:** Suzuki–Miyaura cross-coupling reaction in the presence of a catalytic amount of Fe_3_O_4_@SiO_2_@KIT-6@DTZ-Pd^0^.

Entry	X	R	Time (min)	Yield (%)^a^	M.P (°C)	TON	TOF (h^−1^)
1	I	H	12	95	68–69^40^	177.73	888.65
2	I	4-CH_3_	8	96	46–48^41^	179.607	1347.39
3	I	4-OCH_3_	6	96	86–87^42^	179.607	1796.07
4	Br	4-OH	25	96	162–165^43^	179.607	431.747
5	Br	4-OCH_3_	30	95	88–89^44^	177.73	355.46
6	Br	4-CH_3_	40	94	45–48^41^	175.865	264.062
7	Br	3-OCH_3_	40	94	88–90^31^	175.865	264.062
8	Br	H	55	92	68–70^45^	172.123	187.907
9	Br	4-Cl	70	92	69–71^44^	172.123	147.618
10	Br	4-NO_2_	90	92	113–114^45^	172.123	114.748
11	Cl	H	180	84	67–68^40^	157.156	52.385

^a^Isolated yield.

### Stille cross-coupling reaction

Herein in the final part of this research project, Stille cross-coupling reaction of various aryl halides and triphenyl tin chloride was investigated in the presence of Fe_3_O_4_@SiO_2_@KIT-6@DTZ-Pd^0^. The reaction parameters (including solvent, temperature, type, and amount of base as well as the amount of catalyst) were considered for the cross-coupling of iodobenzene and triphenyl tin chloride. As is evident from Table 5 the highest product yield was obtained in PEG as solvent. Also, the highest yield and the shortest time for mentioned cross-coupling reaction was obtained using K_2_CO_3_ as the base, 6 mg of Fe_3_O_4_@SiO_2_@KIT-6@DTZ-Pd^0^ at 80 °C.

**Table 5:** Optimization of Stille cross-coupling reaction of iodobenzene and triphenyl tin chloride. Significant values are in bold.

Entry	Solvent	Base	Base (mmol)	Catalyst (mg)	Temperature (°C)	Time (min)	Yield (%)^a^
**1**	**PEG**	**K**_**2**_**CO**_**3**_	**3**	**6**	**80**	**35**	**94**
2	DMSO	K_2_CO_3_	3	6	80	35	64
3	DMF	K_2_CO_3_	3	6	80	35	67
4	EtOH	K_2_CO_3_	3	6	Reflux	35	60
5	CH_3_CN	K_2_CO_3_	3	6	80	35	55
6	PEG	KOH	3	6	80	35	75
7	PEG	Na_2_CO_3_	3	6	80	35	71
8	PEG	NaOH	3	6	80	35	68
9	PEG	K_2_CO_3_	1.5	6	80	35	59
10	PEG	K_2_CO_3_	3	7	80	32	94
11	PEG	K_2_CO_3_	3	5	80	35	86
12	K_2_CO_3_	K_2_CO_3_	3	6	100	30	93

^a^Isolated yield.

**Scheme 3:**
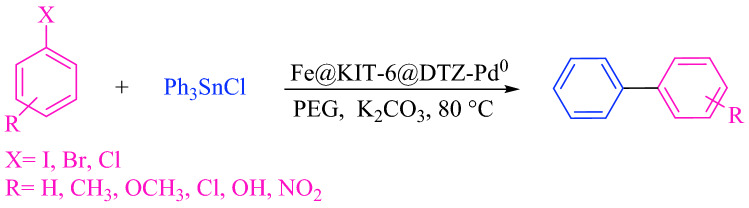
Stille reaction by Fe_3_O_4_@SiO_2_@KIT-6@DTZ-Pd^0^.

To spread out the application of described catalyst in Stille reaction; triphenyl tin chloride reacted with a variety of aryl halides (including chloride, bromide, and iodide) under optimal conditions (Scheme 3). The results including yields and reaction times brought in Table 6.

**Table 6:** Stille cross-coupling reaction in the presence of a catalytic amount of Fe_3_O_4_@SiO_2_@KIT-6@DTZ-Pd^0^.

Entry	X	R	Time (min)	Yield (%)^a^	M.P (°C)	TON	TOF (h^−1^)
1	I	H	35	94	68–69^44^	175.865	301.655
2	I	4-CH_3_	35	92	43–45^44^	172.121	288.819
3	I	4-OCH_3_	20	90	85–86^42^	168.381	505.65
4	Br	4-OH	55	89	162–163^43^	166.510	181.78
5	Br	4-OCH_3_	60	88	84–86^42^	164.639	164.639
6	Br	4-CH_3_	70	85	42–43^43^	159.027	95.454
7	Br	3-OCH_3_	75	87	86–88^31^	162.768	139.596
8	Br	H	100	90	69–70^44^	168.381	101.069
9	Br	4-Cl	110	89	69–70^42^	166.510	90.989
10	Br	4-NO_2_	125	88	112–114^45^	164.639	79.039
11	Cl	H	240	74	68–69^44^	138.447	34.611

^a^Isolated yield.

In order to compare the catalytic performance of Fe_3_O_4_@SiO_2_@KIT-6@DTZ-Pd^0^ with the previously reported catalysts, the results of coupling of phenylboronic acid with iodobenzene by the previously reported methods are shown in Table 7. In this comparison, various parameters such as reaction time, reaction conditions, and efficiency with other catalysts were compared. In this work, the C–C bond formation reaction is performed in ethanol as a green solvent and this catalyst almost shows a shorter reaction time and higher performance than the other catalysts.

**Table 7:** Comparison of Fe_3_O_4_@SiO_2_@KIT-6@DTZ-Pd^0^ with other catalysts for Suzuki–Miyaura reaction using iodobenzene and phenylboronic acid.

Entry	Catalyst	Conditions	Time	Yield (%)	References
1	Fe_3_O_4_@SiO_2_-T-Se/Pd (II)	K_2_CO_3_, EtOH: H_2_O, 60 °C	30 min	95	^46^
2	Pd-AcAc-Am-Fe_3_O_4_@SiO_2_	K_2_CO_3_, DMF: H_2_O, 80 °C	1 h	96	^47^
3	Fe_3_O_4_@SiO_2_‑APTMS‑SAL‑Pd	Na_2_CO_3_, EtOH: H_2_O, 75 °C	15 min	98	^48^
4	GO/Fe_3_O_4_/PAMPS/Pd	K_2_CO_3_, EtOH: H_2_O, 80 °C	2 h	100	^49^
5	Pd/Fe_3_O_4_/r-GO	K_2_CO_3_, H_2_O, reflux	15 min	> 99	^50^
6	Pd/chamomile@Fe_3_O_4_	K_2_CO_3_, EtOH: H_2_O, 60 °C	1 h	96	^51^
7	Fe_3_O_4_@MCM-41@Pd-SPATB	K_2_CO_3_, PEG, 80 °C	25 min	94	^52^
8	Fe_3_O_4_@MCM-41-SB-Pd	K_2_CO_3_, DMF, 120 °C	5 min	98	^53^
9	Fe_3_O_4_@SiO_2_@KIT-6@DTZ-Pd^0^	K_2_CO_3_, EtOH, 75 °C	12 min	95	This work

### Reusability of the catalyst

**Figure 8:**
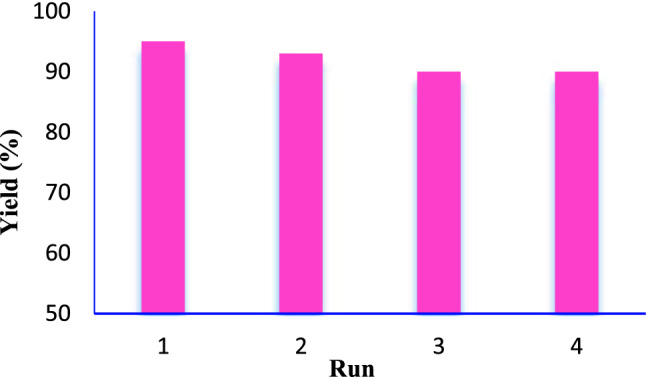
Reusability of Fe_3_O_4_@SiO_2_@KIT-6@DTZ-Pd^0^ in the synthesis of biphenyl.

According to the principles of green chemistry, the recovery of the catalyst at the end of the reaction and its reusability is a highly significant factor. In this aim, Suzuki cross-coupling reaction of iodobenzene and phenylboronic acid was investigated. After each run nanocatalyst was isolated from reaction media by applying a magnet and washed several times with ethanol, then dried and reused for the next experiment. As it can be seen from Fig. 8, the catalyst is recoverable at least up to four runs, with a negligible decrease in its activity.

### Catalyst leaching study

To perform the hot filtration test, the Suzuki reaction was selected as the model reaction and two types of reactions were performed between iodobenzene and phenylboronic acid under optimal reaction conditions. In the first reaction, the biphenyl product was obtained after 6 min (half the reaction time) with a yield of 69. Simultaneously in the second reaction, the same reaction was repeated, but at the half-reaction (after 6 min), the catalyst was removed from the reaction mixture by a magnet and the reaction mixture was allowed to run for another 6 min. The reaction efficiency at this stage was 72%. These experiments confirmed that Fe_3_O_4_@SiO_2_@KIT-6@DTZ-Pd^0^ was necessary to complete the reaction and that confirmed the leaching of palladium during the reaction didn’t occur.

## Conclusions

In this research project, we have introduced a novel magnetic mesoporous material with two unique properties i.e. high porosity and magnetism. These two factors make this nanomaterial an efficient and versatile catalyst. The catalytic activity of Fe_3_O_4_@SiO_2_@KIT-6@DTZ-Pd^0^ was examined in the variety of cross-coupling reactions including Heck, Suzuki, and Stille reactions. These coupling reactions are performed in environmental-friendly conditions with short reaction times and high efficiency and purity of products.

## Experimental

### Preparation of Fe_3_O_4_@SiO_2_@KIT-6

For the synthesis of Fe_3_O_4_@SiO_2_@KIT-6, initially, Fe_3_O_4_@SiO_2_ nanoparticles were synthesized using the previously reported procedure in the literature^54^. In the next step, P123 (1.25 g), Fe_3_O_4_@SiO_2_ (1 g), HCl (37% wt) (2.4 mL), and 1.3 g of *n*-butanol (99.4% wt) were mixed in distilled water (45 mL) and stirred at 35 °C for an hour, then 2.7 g of TEOS was added and the mixture was stirring in the same temperature under nitrogen atmosphere. Finally, the resulting mixture is transmitted into an autoclave and placed in the oven for 24 h at 100 °C. The resulting mixture was washed with 300 mL of ethanol and 20 mL of HCl. Finally, Fe_3_O_4_@SiO_2_@KIT-6 was calcified at 550 °C for 6 h.

### Preparation of Fe_3_O_4_@SiO_2_@KIT-6@DTZ

In a 100 mL balloon, Fe_3_O_4_@SiO_2_@KIT-6 (1 g) sonicated for 30 min in toluene (25 mL), then, 1.5 mL of 3-chloropropyltriathoxycylanine (CPTES) was added and the resulting mixture was stirred for 24 h at 90 °C under nitrogen atmosphere. The obtained solid material was washed with dichloromethane (50 mL). Subsequently, Fe_3_O_4_@SiO_2_@KIT-6@CPTES (1 g) was dispersed in 25 mL of DMF by sonication for 30 min, which was followed by adding 3 mmol of dithizone (diphenylthiocarbazone) and 3.5 mmol triethylamine. The resulting mixture was stirred for 48 h at 100 °C with. The obtained precipitate was then washed several times by organic solvents and dried at room temperature.

### Preparation of Fe_3_O_4_@SiO_2_@KIT-6@DTZ-Pd^0^

In a 100 mL round-bottom flask Fe_3_O_4_@SiO_2_@KIT-6@DTZ (1 g) dispersed in 50 mL ethanol via sonication for 20 min. Then, 0.5 g of Pd(OAc)_2_ was added to the mixture and stirred for 20 h, at reflux conditions under nitrogen atmosphere. Finally, NaBH_4_ (0.6 mmol) was added to the reaction mixture and stirred under the same conditions for 2 more hours. Then, the reaction mixture cooled down to room temperature, and magnetic mesoporous material (Fe_3_O_4_@SiO_2_@KIT-6@DTZ-Pd^0^) was isolated by a magnet and washed several times with ethanol.

### General procedure for Mizoroki–Heck cross-coupling reaction

To perform Heck reaction, in a 5 mL round bottom flask, aryl halide (1 mmol), butyl acrylate (1.2 mmol), 3 mmol of potassium carbonate, and Fe_3_O_4_@SiO_2_@KIT-6@DTZ-Pd^0^ (5 mg) were added to 2 mL of PEG and the mixture stirred at 100 °C. The progress of the reaction was followed by TLC. After completion of cross-coupling reaction, 10 mL water was added and catalyst separated by an external magnet. The corresponding product was extracted by ethyl acetate from a mixture of water and ethyl acetate.

#### n-Butyl cinnamate

mp: Oil (lit.ref Oil); ^1^H NMR (300 MHz, CDCl_3_): δ 7.67–7.72 (d, J = 7.69, 1H, alkene), 7.52 (d, J = 7.52, 2H, ArH), 7.39–7.40 (t, J = 7.39, 2H, ArH), 7.37–7.38 (t, J = 7.38, 1H, ArH), 6.43–6.48 (d, J = 4.55, 1H, alkene), 4.22 (t, J = 4.22, 2H, CH2), 1.70 (m, J = 1.68, 2H, CH2), 1.43–1.46 (m, J = 1.44, 2H, CH2), 0.96 (t, J = 0.97, 3H, CH3) ppm (Fig. S1).

### General procedure for Suzuki–Miyaura cross-coupling reaction

To a 5 mL round bottom flask, aryl halide (1 mmol), phenylboronic acid (1 mmol), potassium carbonate (2.5 mmol), catalyst (5 mg), and 2 mL of ethanol solvent were added. The reaction mixture was stirred for a specified time at 75 °C. After reaction completion, the catalyst was separated using an external magnet and the reaction mixture was transferred to a separating funnel and extracted with ethanol. The extracted organic phase was collected and dried. The obtained product was then spectrally analyzed after purification.

#### 4-Methyl-1,1′-biphenyl

mp: 45–48 °C (lit.ref 50–51 °C); TLC (n-hexane); ^1^H NMR (500 MHz, DMSO-d6) δ 7.62 (d, J = 7.63, 2H, ArH), 7.54 (d, J = 7.55, 2H, ArH), 7.44 (t, J = 7.44, 2H, ArH), 7.33 (t, J = 7.33, 1H, ArH), 7.27 (d, J = 7.26, 2H, ArH), 2.34 (s, 3H, CH_3_) ppm (Fig. S2).

#### 4-Nitrobiphenyl

mp: 113–114 °C (lit.ref 103–106 °C); TLC (n-hexane); ^1^H NMR (300 MHz, CDCl_3_) δ 8.29–8.30 (d, J = 8.30, 2H, ArH), 7.72–7.76 (d, J = 7.74, 2H, ArH), 7.62–7.63 (d, J = 7.62, 2H, ArH), 7.51 (t, J = 7.52, 2H, ArH), 7.48 (t, 1H, ArH) ppm (Fig. S3).

### General procedure for Stille cross-coupling reaction

In a 5 mL round bottom flask, aryl halide (1 mmol), triphenyl tin chloride (0.5 mmol), potassium carbonate (3 mmol), catalyst (6 mg), and 2 mL of PEG solvent were added. The reaction mixture was stirred for a specified time at 80 °C. The reaction progress followed by TLC. After the reaction completion, the catalyst was separated by an external magnetic field, and the mixture was transferred to a separating funnel and extracted with ethyl acetate and water. The obtained organic phase was collected and dried. The obtained product was then spectrally analyzed after purification.

#### [1,1′-Biphenyl]-4-ol

mp: 162–163 °C (lit.ref 161–164 °C); TLC (n-hexane); ^1^H NMR (500 MHz, DMSO-d6) δ 9.60 (s, 1H, OH), 7.56 (d, J = 7.56, 2H, ArH), 7.48 (d, J = 7.49, 2H, ArH), 7.39 (t, J = 7.39, 2H, ArH), 7.26 (t, J = 7.26, 1H, ArH), 6.88 (d, J = 6.89, 2H. ArH) ppm (Fig. S4).

#### 4-Chlorobiphenyl

mp: 69–70 °C (lit.ref 70–72 °C); TLC (n-hexane); ^1^H NMR (500 MHz, DMSO-d6): δ 7.68 (d, J = 7.67, 2H, ArH), 7.66 (d, J = 7.64, 2H, ArH), 7.51 (d, J = 7.5, 2H, ArH), 7.46 (t, J = 7.46, 2H, ArH), 7.38 (t, J = 7.38, 1H, ArH) ppm (Fig. S5).

## Supplementary Information

Supplementary Information.
