# Supplementary material for: Design and synthesis of Fe3O4@SiO2@KIT-6@DTZ-Pd0 as a new and efficient mesoporous magnetic catalyst in carbon–carbon cross-coupling reactions
Source: Sci Rep. 2021 Dec 14;11:23967. doi: 10.1038/s41598-021-03485-4 (PMC8671489; doi:10.1038/s41598-021-03485-4)
Supplement: Supplementary file 1 — Supplementary Information. [file 41598_2021_3485_MOESM1_ESM.docx]

Design and synthesis of Fe_3_O_4_@SiO_2_@KIT-6@DTZ-Pd^0^ as a new and efficient mesoporous magnetic catalyst in carbon-carbon cross-coupling reactions

Zahra Moradi ^1^, Arash Ghorbani-Choghamarani ^2,^*

^1^ Department of Chemistry, Faculty of Sciences, Ilam University, P.O. Box 69315516, Ilam, Iran. E-mail address: zahra.m419@yahoo.com or z.moradi@ilam.ac.ir.
^2^ ^*^Corresponding author: Ghorbani-Choghamarani, Department of Organic Chemistry, Faculty of Chemistry, Bu-Ali Sina University, P.O. Box 6517838683, Hamedan, Iran; E-mail address: a.ghorbani@basu.ac.ir or arashghch58@yahoo.com

**1. General procedure for cross-coupling reactions by Fe_3_O_4_@SiO_2_@KIT-6@DTZ-Pd^0^**

**1.1. General procedure for Mizoroki–Heck cross-coupling reaction**

To conduct Heck reaction in a round bottom balloon, 5 ml of aryl halide (1 mmol), alkene (1.2 mmol) and 3 mmol of potassium carbonate were added with 5 mg of Fe_3_O_4_ catalyst in 2 ml of PEG and stirred at 100 °C. The reaction was followed by TLC in n-hexane tank solvent until the reaction was completed and then, the nanoparticles and inorganic materials were separated by a magnet using water and ethyl acetate and after purification were spectrally analyzed.

**n-Butyl cinnamate.** mp: Oil (lit.ref Oil); ^1^H NMR (300 MHz, CDCl_3_): δ 7.67-7.72 (d, J=7.69, 1H, alkene), 7.52 (d, J=7.52, 2H, ArH), 7.39-7.40 (t, J=7.39, 2H, ArH), 7.37-7.38 (t, J=7.38, 1H, ArH), 6.43-6.48 (d, J=4.55, 1H, alkene), 4.22 (t, J=4.22, 2H, CH_2_), 1.70 (m, J=1.68, 2H, CH_2_), 1.43-1.46 (m, J=1.44, 2H, CH_2_), 0.96 (t, J=0.97, 3H, CH_3_) ppm (**Figure S1**).

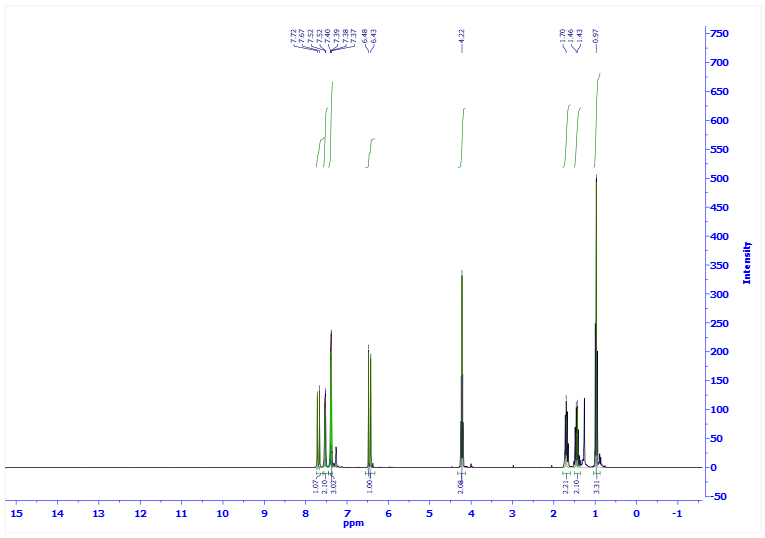
 **Figure S1**. ^1^H NMR spectrum of n-butyl cinnamate.

**1.2. General procedure for Suzuki–Miyaura cross-coupling reaction**

In a 5 ml round bottom balloon, aryl halide (1 mmol), phenyl boronic acid (1 mmol), potassium carbonate as base (2.5 mmol), catalyst (5 mg) and 2 ml of ethanol solvent were added. The reaction mixture was stirred for a specified time at 75 °C. After reaction was completed, the catalyst was separated using an external magnetic field and the reaction mixture was transferred to a separating funnel and extracted with ethanol. The extracted organic phase was collected and dried. The obtained product was then spectrally analyzed after purification.

**4-Methyl-1,1'-biphenyl.** mp: 45-48 °C (lit.ref 50-51 °C); TLC (n-hexane); ^1^H NMR (500 MHz, DMSO-d6) δ 7.62 (d, J=7.63, 2H, ArH), 7.54 (d, J=7.55, 2H, ArH), 7.44 (t, J=7.44, 2H, ArH), 7.33 (t, J=7.33, 1H, ArH), 7.27 (d, J=7.26, 2H, ArH), 2.34 (s, 3H, CH_3_) ppm (**Figure S2**).

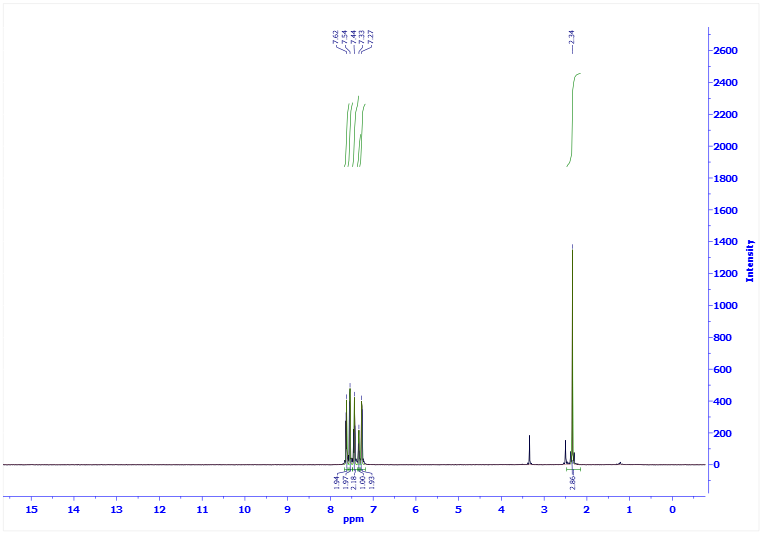
 **Figure S2**. ^1^H NMR spectrum of 4-methyl-1,1'-biphenyl.

**4-Nitrobiphenyl.** mp: 113-114 °C (lit.ref 103-106 °C); TLC (n-hexane); ^1^H NMR (300 MHz, CDCl_3_) δ 8.29-8.30 (d, J= 8.30, 2H, ArH), 7.72-7.76 (d, J= 7.74, 2H, ArH), 7.62-7.63 (d, J= 7.62, 2H, ArH), 7.51 (t, J= 7.52, 2H, ArH), 7.48 (t, 1H, ArH) ppm (**Figure S3**).

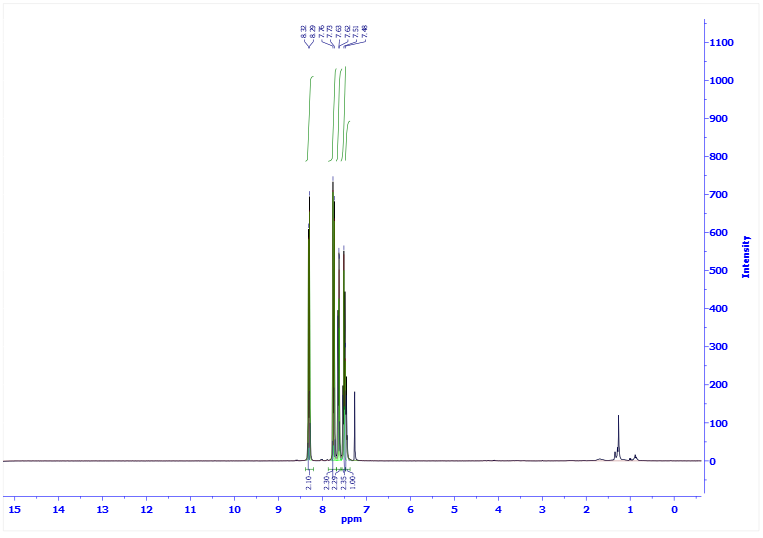
 **Figure S3**. ^1^H NMR spectrum of 4-nitrobiphenyl.

**1.3. General procedure for Stille cross-coupling reaction**

In a 5 ml round bottom balloon, aryl halide (1 mmol), triphenyl tin chloride (0.5 mmol), potassium carbonate as base (3 mmol), catalyst (6 mg) and 2 ml of PEG solvent were added. The reaction mixture was stirred for a specified time at 80 °C. The reaction progression was followed by TLC in n-hexane tank solvent. After the reaction completion, the catalyst was separated by an external magnetic field and its mixture was transferred to a separating funnel and extracted with ethyl acetate and water. The obtained organic phase was collected and dried. The obtained product was then spectrally analyzed after purification.

**[1,1'-Biphenyl]-4-ol.** mp: 162-163 °C (lit.ref 161-164 °C); TLC (n-hexane); ^1^H NMR (500 MHz, DMSO-*d6*) δ 9.60 (s, 1H, OH), 7.56 (d, J=7.56, 2H, ArH), 7.48 (d, J=7.49, 2H, ArH), 7.39 (t, J=7.39, 2H, ArH), 7.26 (t, J=7.26, 1H, ArH), 6.88 (d, J=6.89, 2H. ArH) ppm (**Figure S4**).

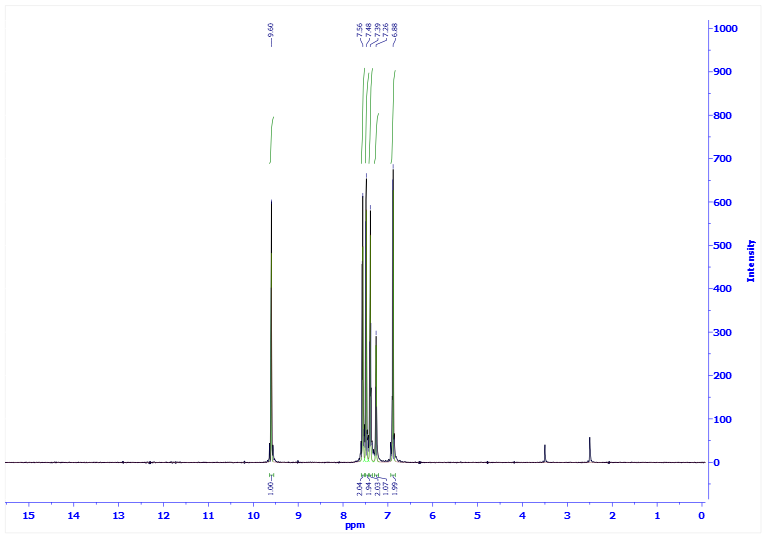
 **Figure S4**. ^1^H NMR spectrum of [1,1'-biphenyl]-4-ol.

**4-Chlorobiphenyl.** mp: 69-70 °C (lit.ref 70-72 °C); TLC (n-hexane); ^1^H NMR (500 MHz, DMSO-*d6*): δ 7.68 (d, J=7.67, 2H, ArH), 7.66 (d, J=7.64, 2H, ArH), 7.51 (d, J=7.5, 2H, ArH), 7.46 (t, J=7.46, 2H, ArH), 7.38 (t, J=7.38, 1H, ArH) ppm (**Figure S5**).

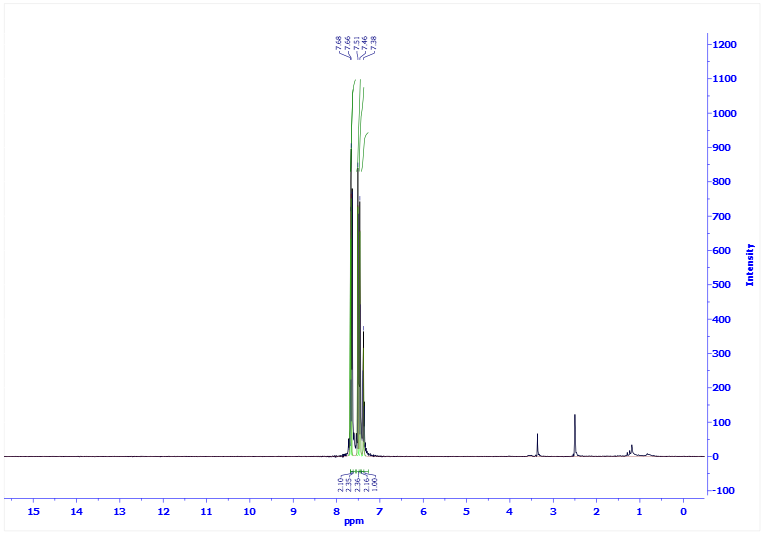


**Figure S5**. ^1^H NMR spectrum of 4-chlorobiphenyl.
